# Data-driven head model individualization from digitized electrode positions or photogrammetry improves M/EEG source localization accuracy

**DOI:** 10.1162/IMAG.a.1073

**Published:** 2026-01-08

**Authors:** Nils Harmening, Alexander von Lühmann, Benjamin Blankertz

**Affiliations:** BIFOLD – Berlin Institute for the Foundations of Learning and Data, Berlin, Germany; Intelligent Biomedical Sensing (IBS) Lab, Machine Learning Department, Technical University of Berlin, Berlin, Germany; Neurotechnology Lab, Technical University of Berlin, Berlin, Germany

**Keywords:** EEG, MEG, inverse problem, source localization, head modeling, BEM, photogrammetry

## Abstract

We propose a data-driven algorithm to approximate individual head anatomies to improve source localization accuracy over the widely used standard head models Colin27 and ICBM-152 when structural MRI/CT scans are not available. Based on a low-dimensional representation of a large head model database, we derive individual head shape parameters solely from additional knowledge of the subject’s scalp, which is obtained, for example, from photogrammetry scans or precise electrode positions. We demonstrate in an experimental study of 16 subjects that our approach provides better-approximated head model anatomies than other existing approaches, even when using scalp proxies derived from a smartphone scan. Moreover, in an EEG simulation study involving 22 heads, we show that our head models outperform standard and other individualization approaches in terms of source localization accuracy. As our proposed head model individualization method does not require structural scans of each subject, it can help improve source localization with minimal effort in future M/EEG studies, particularly when MRI/CT scans are not available.

## 1 Introduction

### 1.1 Motivation

Magnetoencephalography (MEG) and electroencephalography (EEG) are non-invasive methods for measuring neural activity on the human scalp and in other species. Both MEG and EEG, referred to as M/EEG in the following, record a spatially smeared linear mixture of neural sources that are active in parallel (Haufe et al., 2014). The disentangling of different signals at the sensors is commonly done by (blind) source separation techniques (Blankertz et al., 2011; Makeig et al., 1996), which all introduce different assumptions to constrain the solution space.

The most frequently used techniques to localize the origin of such disentangled signals compare the potentials at the electrodes with those computed by a so-called anatomical head model. Head models are used to model empirically known electrical tissue properties to solve the forward M/EEG problem, that is, the relevant quasistatic Maxwell equations for cortical M/EEG sources. Although introducing further inaccuracies, equivalent current dipoles (ECDs) are an efficient approximation for modeling synchronously firing and parallel-oriented bunches of adjacent neurons (de Munck et al., 1988).

For realistic head shapes, the differential head model equations can only be solved numerically. A prominent method is the boundary element method (BEM), in which the differential equations are transformed into integral equations defined only on surfaces of different tissue compartments (Kybic et al., 2005). Simplifying the head by using nested tissue compartments, such as the scalp, skull, cerebrospinal fluid (CSF), and cortex, means that the volume conductor is piecewise homogeneous and isotropic, which is a necessary assumption for BEM, and, at the same time, for most tissues physiologically acceptable (Vorwerk et al., 2014).

Assuming tissue isotropy, the accuracy of BEM is comparable to that of models with higher anatomical accuracy, such as finite element methods (FEM) (Acar & Makeig, 2010; Oostenveld & Oostendorp, 2002). To additionally account for tissue anisotropy in FEM, extra information is needed, such as from diffusion tensor imaging (DTI), which is often unavailable.

Besides the anatomical tissue simplification and the equivalent current dipole approximation, several additional factors influence the accuracy of forward models, and consequently, the accuracy of inverse problem solutions. These are most notably the conductivity values and especially the skull/brain conductivity ratio (Acar & Makeig, 2013; Goncalves et al., 2003), the degree of discretization (triangulation grid-size) (Acar & Makeig, 2010), and the skull anisotropy (Dannhauer et al., 2011; Wolters et al., 2006).

However, the two most influential causes of source localization inaccuracies are imprecise electrode positions (Homölle & Oostenveld, 2019; Khosla et al., 1999) and the usage of template or average head models when the subject’s structural MRI or CT scans are unavailable (Acar & Makeig, 2013). Popular template head models are the ICBM-152, an average of co-registered individual MRIs of 152 subjects provided by the Montreal Neurological Institute (MNI) (Fonov et al., 2009, 2011) and the finer detailed Colin27, an average of 27 MRIs from a single individual (Colin Holmes), also in the MNI coordinate system (Holmes et al., 1998).

While researchers increasingly measure and digitize exact electrode positions for improved source localization by using photogrammetry (Clausner et al., 2017), structured-light 3D scanners (Homölle & Oostenveld, 2019), electromagnetic technologies like the widely used Polhemus FASTRAK (Jaiswal et al., 2023; Mosher & Funke, 2020), or other hardware solutions (Shirazi & Huang, 2019), individual MRI or CT images often remain too costly and time-consuming for every subject, especially for large-scale studies. The majority then uses the mentioned standard head models, although Colin27 and the ICBM-152 might not be representative for a large part of the target population.

Several studies have repeatedly emphasized the importance of using realistic volume conductor models constructed from individual subject anatomies for obtaining accurate forward and inverse model solutions (Acar & Makeig, 2013; Baillet et al., 2001; Hämäläinen et al., 1993; Oostenveld & Oostendorp, 2002; von Ellenrieder et al., 2009; Vorwerk et al., 2014). Therefore, in the frequent case when structural scans are not available, it is of great importance to reduce the shape differences between the individual subjects’ heads and those used for head modeling to improve M/EEG source localization performance.

### 1.2 Approaches so far

Only a few studies have so far attempted to approximate individual head shapes for volume conduction head models in the absence of ground-truth head anatomies.

Chronologically, the first work in this direction was by Leahy et al. (1998), who fitted three concentric spheres to the local curvatures of the scalp and outer and innermost skull as derived from a CT scan. Gibson et al. (2003) warped a generic adult head surface to a sparse set of measured individual surface points and generated a finite element mesh of one tissue type from the warped surface. However, these early approaches lack either relevant realistic head anatomies or the critical distinction between different biological tissues.

Darvas et al. (2006) then warped the scalp surface mesh of the average ICBM-152 head to measured sensor locations using a thin plate spline (TPS) warp and applied the same warp to the remaining surface meshes. This algorithm was implemented in the Neuroelectromagnetic Forward Head Modeling Toolbox (NFT) (Acar & Makeig, 2010) and the warped 4-shell ICBM-152 BEM (referred to as “wMNI-4”) was shown to outperform standard head model templates (Acar & Makeig, 2013). Similarly, Rhodes et al. (2025) also warped a template MRI to the scalp surface, but used a simpler affine transform applied directly to the template MRI. On sensory-task OPM data from 20 subjects, they showed that this warped template MRI produced an average source localization difference of about 2.75 mm compared to using each subject’s actual MRI. All those attempted shape improvements have, however, by definition, the drawback of inheriting the details of the template, although these details might still differ considerably from those of a single individual.

Hansen et al. (2016) derived a low-dimensional parametrization of 16 head geometries and various tissue conductivities using Principal Component Analysis (PCA). In the next step, the individual shape parameters were estimated directly when solving the inverse problem using only the recorded EEG data. Unfortunately, this idea directly works on leadfields and is thus restricted to the same sourcemodel positions and 70 electrode locations.

Valdés-Hernández et al. (2009) used a description of the cortical surface with spherical harmonics to improve EEG source imaging through different averaging approaches over many heads. However, it was not tried to individualize the head models by, for example, using additional information such as scalp surface or electrode positions. The best result was achieved by directly averaging leadfields, but this restricts usage to the same montage and predefined source points as in Hansen et al. (2016).

### 1.3 Our approach: PCAwarp

**Fig. 1.**
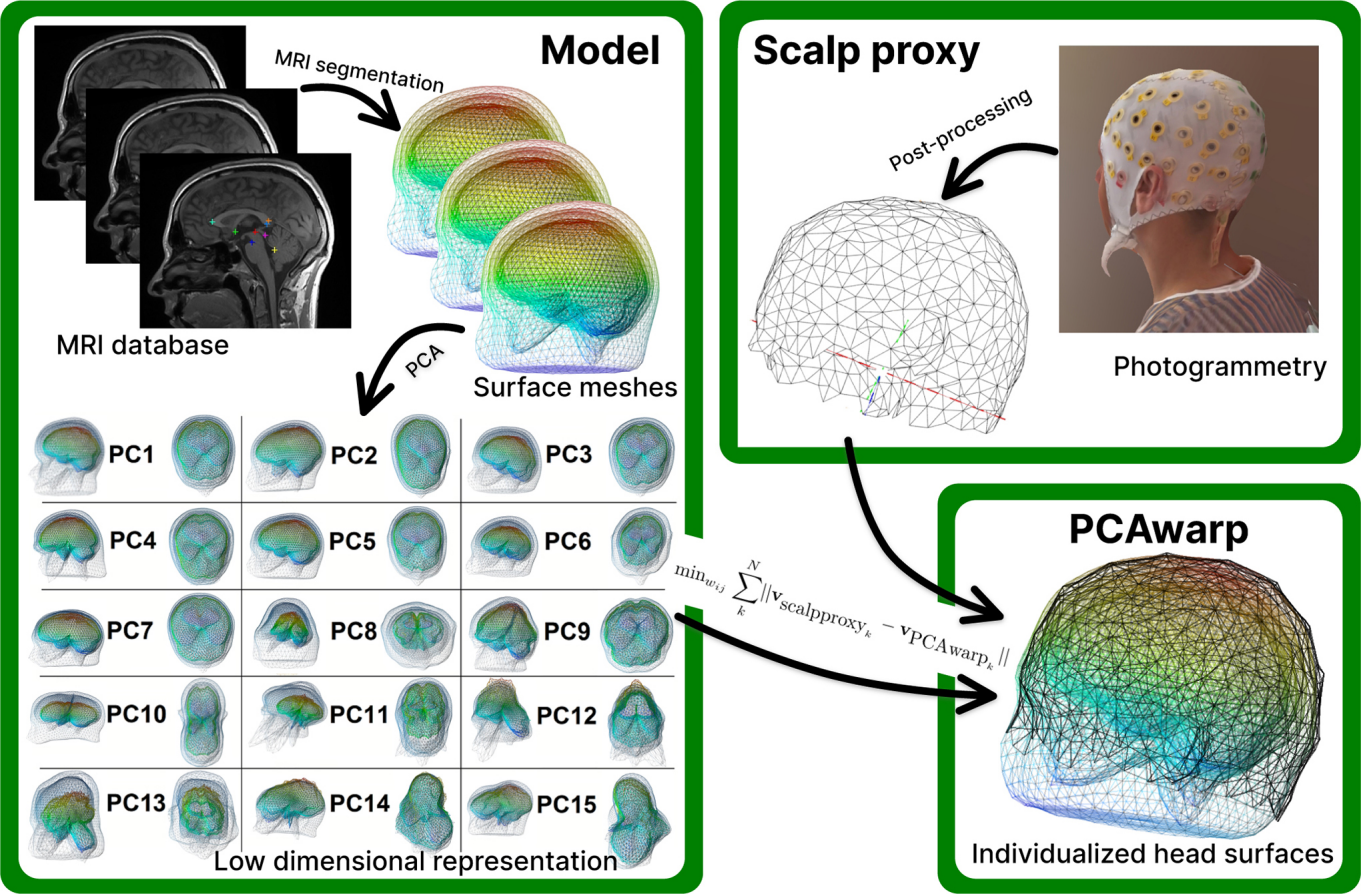
Overview of the PCAwarp approach. A PCA gains a low-dimensional representation of the surface meshes based on a large database of automatically segmented and consistently meshed MRI scans (left). To compute individual head model surfaces (bottom right), a scalp proxy consisting of a few points distributed over the entire upper head, as obtained from a photogrammetry scan, is required (top right). The distances between all scalp proxy points ${\text{v}}_{\text{scalpproxy}}$ and the PCAwarp scalp surface vertices ${\text{v}}_{\text{PCAwarp}}$ are minimized by adjusting the PCAwarp parameters ($w_{ij}$ ). In doing so, the remaining surfaces (skull, CSF, cortex) are automatically estimated.

Our approach is based on a low-dimensional representation of all four surface meshes of a BEM head model and builds upon the idea presented in Miklody et al. (2017). It utilizes the PCA’s principal components (PCs) (Pearson, 1901) trained on 316 head model anatomies to fit any scalp proxy, whether from photogrammetry scans, precisely measured electrode positions, or any other scalp digitization. Figure 1 summarizes the general idea and the single steps of our approach, and the following Section 2 explains our algorithm in more detail.

In Section 3, we demonstrate that our approach already provides better-approximated head model anatomies when using photogrammetry scans and simple electrode digitization via a smartphone scan, than other existing approaches. Moreover, we show in a simulation study that our approach outperforms standard head models and existing individualization approaches in terms of source localization accuracy.

As our proposed head model individualization method does not require structural scans of each individual subject, it has the potential to improve source localizations with little effort in many future M/EEG studies. In the following, we will refer to our approach as “PCAwarp”.

The complete source code and a detailed usage description are publicly available at https://github.com/harmening/headmodel_individualization. A practitioner’s guide demonstrates how to perform a photogrammetry scan using a smartphone and a free scanning app in under 10 min, and how to integrate the scan into the PCAwarp algorithm and export to various formats and common toolboxes like FieldTrip, MNE-python. Further inputs from common sensor digitization solutions, like Polhemus FASTRAK, BrainVision CapTrak, are supported. Note that the technical derivations in this paper were required to develop and evaluate the methodology. A deeper understanding of those is not needed for the application.

## 2 Methods

Figure 1 summarizes the main steps of the PCAwarp method, explained in the following in more detail.

### 2.1 Constructing a head model database

A large database of head shapes was constructed for data-driven approximation of individual head shapes based on knowledge of the individual scalp surface or any proxy, such as exact electrode positions.

#### 2.1.1 MRI database

For the collection of the large head model database, we used an open-access cross-sectional MRI database, namely the Open Access Series of Imaging Studies 1 (OASIS1) (Marcus et al., 2007). Considering only healthy subjects, the study comprises 316 male and female participants aged 18 to 96, recruited from Washington University and the Washington University Alzheimer’s Disease Research Center (ADRC). The co-registered average of 3 to 4 MRI scans of each participant with 1 mm resolution was used for segmentation.

#### 2.1.2 Automatic segmentation

To derive surface meshes from the individual MRI scans, which are constructed as technically similar as possible, an automatic segmentation pipeline (Harmening & Miklody, 2022) was developed. It includes the following steps:

##### 2.1.2.1 Landmark detection and coordinate system alignment

All meshes need to be aligned to the same coordinate system. The *acpcdetect* of the Automatic Registration Toolbox (ART) (Ardekani & Bachman, 2009) automatically detects the anterior and posterior commissures (AC and PC) landmarks and the mid-sagittal plane. It also directly translates the input MRI into the ACPC coordinate system, which retains its original size, i.e., it is not normalized to a template, has its origin at AC, and is RAS-oriented (x = Right, y = Anterior, z = Superior).

##### 2.1.2.2 Segmentation by SPM12 with eTPM.nii

The posterior probability of each MRI voxel belonging to a specific tissue type (scalp, skull, CSF, gray matter (GM), white matter (WM)) is estimated by the *Unified Segmentation* framework of Ashburner and Friston (2005) as implemented in Statistical Parametric Mapping (SPM) 12 software package (*new_segment* in SPM8). The neck-extended tissue probability map template *eTPM.nii* developed by Huang et al. (2013) is used as prior probability distribution template. The calculated probability distributions are converted into binary masks for each tissue, followed by smoothing with a Gaussian low-pass filter using *Andy’s tools* (Huang et al., 2013). The following automatic correction of obvious morphological errors includes assigning unassigned voxels and reclassifying GM voxels that are directly adjacent to skull voxels as CSF voxels to avoid potential CSF discontinuities. To correct further morphological errors, the CSF and skull surfaces were broadened to a minimum thickness of three voxels and the scalp to a minimum thickness of four voxels.

Next, GM and WM are combined into a single tissue type, ‘cortex’, a common practice in BEM modeling (Vorwerk et al., 2012).

##### 2.1.2.3 Mesh construction by FieldTrip

Boundary surface meshes per tissue are triangulated with *projectmesh* as implemented in *triangulate_seg* in the FieldTrip toolbox (Oostenveld et al., 2011). This triangulation algorithm starts at the center of the segmented volume and projects the 1922 vertices of an evenly triangulated sphere onto the outer surface. The resulting surface is, by construction, star-shaped from the origin of the sphere. The final surface meshes share the same triangulation, meaning that the same vertices are positioned similarly for every head. Ten out of 1264 surface meshes (4 tissues of the 316 healthy OASIS1 heads) needed further (manual) correction^1^. A further Laplace flow mesh smoothing is applied to the cortex mesh for more stable BEM simulations.

##### 2.1.2.4 Sourcemodel and electrodes

For the later EEG simulations, equivalent current dipole positions were calculated at a 2/3 distance between the gray and white matter surface using the accurate cortex segmentation and cortical thickness determination of the Computational Anatomy Toolbox (CAT) (Gaser et al., 2023). The equivalent current dipoles are oriented according to their outward-pointing mesh normals to match the pyramidal cells’ major orientation perpendicular to the cortical surface (Buzsáki et al., 2012).

The 64 locations from the EEG 10-10 standard were aligned using SPM’s nonlinear, voxel-wise transformation mapping from the eTPM.nii to the individual head. An additional projection to the scalp mesh’s closest point was applied to ensure that the electrodes are in contact with the scalp surface mesh.

##### 2.1.2.5 Transformation to fiducial-based coordinate systems

The same procedure as for the electrodes was applied to the fiducials nasion (NAS) and left and right pre-auricular (LPA and RPA). By using these individual fiducials, all meshes were transformed into the CTF coordinate system, which retains its original size, has its origin strictly between LPA and RPA, with the x-axis pointing toward NAS, and is ALS-oriented (x = Anterior, y = Left, z = Superior). The final head database is in the CTF coordinate system since every unknown subject can be brought into this system by manually determining the fiducials NAS, LPA, and RPA.

### 2.2 Deriving a low-dimensional representation of head shapes

Due to the consistent segmentation and meshing algorithms, each vertex i of every head is approximately at the same position in the CTF coordinate system. This results in four surface meshes (cortex, CSF, skull and scalp) for each head, comprising 1922 vertices with three coordinates, that is, 23064 coordinates. Adding NAS, LPA, and RPA, we end up with 316 observations of 23073 coordinates.

**Fig. 2.**
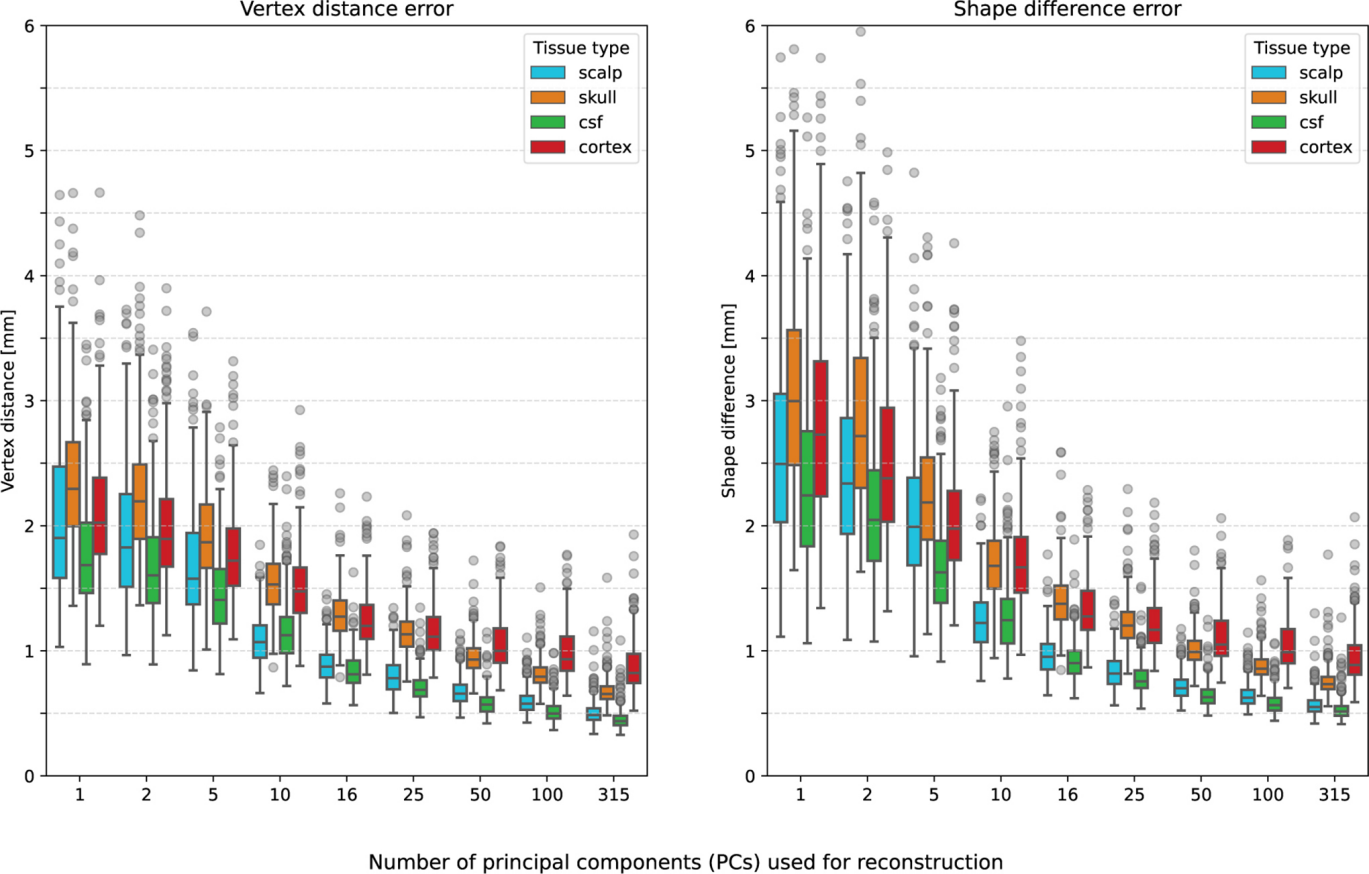
Head model approximation error on unseen subjects in mm using different numbers of PCs of the low-dimensional representation for reconstruction. A PCA built the low-dimensional representation of all heads except the one on which the approximation error was computed (leave-one-out cross-validation). The left plot measures the shape approximation error in vertex distance, that is, the exact distance between the corresponding vertices, while the right plot uses the shape difference as an error metric, which is approximately the Euclidean distance along the surface mesh normals.

A PCA of the normalized coordinates extracts the main shape components and the first few PCs are already sufficient to reduce the mean approximation error of an unseen head to below 1.5  mm. This is shown in Figure 2 in two different shape difference metrics (introduced in the next section). In a leave-one-head-out cross-validation, it is shown how well a reconstruction of an unseen sample (not included in training data) is possible based on different numbers of first PCs used for reconstruction. The reconstruction was solved using linear regression, with the sum of the vertex distances as the cost function. Setting a precision threshold at 1 mm, we found that using 16 out of 315 PCs is the minimum number to meet that criterion. Moreover, one can see that the scalp and cortex are fitted best, while the more complicated surface meshes of the skull (different segmentations in the inferior region) and cortex (different gyri/sulci) are reconstructed less accurately.

### 2.3 Approximating individual head geometry based on scalp proxies

The low-dimensional representation can be used to approximate the four surface meshes of an individual subject based solely on knowledge of the scalp geometry, as explained in this section.

#### 2.3.1 Metrics

Let some scalp mesh proxy be given by precisely measured electrode positions or photogrammetry scans of the subject’s head. Four surface meshes (scalp, skull, CSF, cortex) and the three fiducials 4⋅1922+3=7691 vertices are constructed by choosing PC weights $w_{ij}$ with i=1,…,7691 and j=1,…,316 via

$$\begin{array}{c} {\text{v}}_{i}^{PCAwarp}=\sum_{j=1}^{316}w_{ij}{\text{PC}}_{j}. \end{array}$$ (1)

The aim is to minimize the distance between the measured scalp proxy vertices ${\text{v}}_{\text{scalpproxy}}$ and the fitted scalp mesh from the PCA reconstruction by adjusting the PC weights $w_{ij}$ :

$$\begin{array}{c} {\text{min}}_{w_{ij}}\sum_{k=1}^{N}\left\|\;{\text{v}}_{{\text{scalpproxy}}_{k}}-{\text{v}}_{{\text{PCAwarp}}_{k}}\right\| \end{array}$$ (2)

**Fig. 3.**
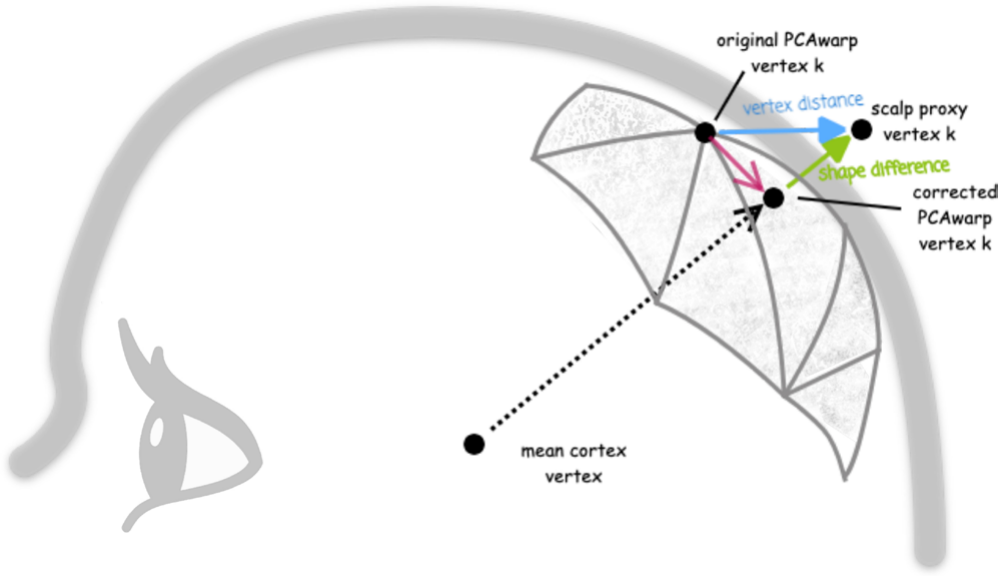
Schematic representation of the two error metrics during the PCA warping procedure for a single scalp proxy vertex k. By adjusting the PC weights four surface meshes including the scalp mesh (sketched with grey-shaded triangles) are constructed. The distance of the closest mesh vertex (*original PCAwarp vertex k*) to the *scalp proxy vertex k* (blue vector) is subject to minimization using the metric *vertex distance*. By using the more appropriate metric *shape difference*, the distance between the *scalp proxy vertex k* and the mesh intersection of the (dotted) raytracing (*corrected PCAwarp vertex k*) is minimized (green vector). Note that both metrics only measure the error according to the scalp mesh. The other three surfaces and the fiducials are scaled according to the PC weights and the database dependencies.

Here, N is the number of vertices of the scalp proxy and ${\text{v}}_{\text{PCAwarp}}$ is a set of vertices on the PCAwarp scalp mesh. Following Miklody et al. (2017), to each vertex k of the scalp proxy, the closest vertex on the PCAwarp scalp mesh ${\text{v}}_{{\text{PCAwarp}}_{k}}$ is found and added as k-th element to the set of vertices ${\text{v}}_{\text{PCAwarp}}$ . However, this might not accurately reflect the closeness of the scalp proxy and the fitted scalp, as it includes errors in all three dimensions (schematically shown by the blue vector in Fig. 3). A better metric is to measure the error only orthogonal to the mesh surface (shape difference), so approximately along a surface mesh normal intersecting with the scalp proxy point (green vector in Fig. 3), because it is independent of the meshing algorithm, i.e., the positions of vertices on the surface mesh. Since the surface normals are sometimes unreliable in case of distorted meshes during the optimization procedure, the vector pointing from the mean cortex vertex in the middle of the head to the scalp proxy vertex is used instead.

In the following, we refer to the first metric as *vertex distance* because it measures the exact Euclidean distance between the corresponding vertices and to the second metric as *shape difference*, as it tries to acquire the actual tissue shell distance and reflects the closeness of a point cloud to a surface mesh better. Both metrics are represented in the objective function in eqn. 2. Still, the set of vertices ${\text{v}}_{\text{PCAwarp}}$ differs: The vertex distance uses the vertices of the PCAwarp scalp mesh closest to the scalp proxy points. At the same time, the shape difference replaces this set of PCAwarp scalp mesh vertices by the corresponding intersections of the mentioned vector with the PCAwarp scalp mesh.

Technically, the shape difference is computed by first defining the vector starting at the mean of all cortex vertices pointing toward the currently evaluated scalp mesh proxy vertex k. Second, the intersection of this vector with the fitted scalp mesh is computed (*raytracing*) and used as ${\text{v}}_{{\text{PCAwarp}}_{k}}$. Its Euclidean distance to ${\text{v}}_{{\text{scalpproxy}}_{k}}$ then comprises the final error measure for the currently evaluated vertex k and is repeated for all N vertices. Note that this means that the set of vertices on the PCAwarp scalp mesh ${\text{v}}_{\text{PCAwarp}}$ gets recomputed in each optimization step. Figure 3 schematically summarizes this PCA warping procedure for a single vertex k.

#### 2.3.2 Regularization

An additional penalty term g can be added to this error measure to penalize overly close surface meshes. This ensures that the final nested meshes are still useful for BEM head modeling and do not intersect. The penalty term g is constructed such that for all pairs of vertices of all mesh combinations of $ℳ=\{m_{\text{scalp}},\;m_{\text{skull}},\;m_{\text{CSF}},\;m_{\text{cortex}}\}$ the Euclidean distance is computed. If this distance is smaller than a threshold T (set to T=1 mm), the threshold T minus the distance is summed up to the penalty term:

$$g=\sum_{m_{1}\in M}\sum_{\underset{m_{1}\neq m_{2}}{m_{2}\in M}}\sum_{p}^{N_{m1}}\sum_{q\neq p}^{N_{m2}}\max(0,T||v_{m1p}-v_{m2q}||)$$ (3)

The final minimization task

$$\begin{array}{c} {\text{min}}_{w_{ij}}\sum_{k=1}^{N}\left\|{\text{v}}_{{\text{scalpproxy}}_{k}}-{\text{v}}_{{\text{PCAwarp}}_{k}}\right\|\;\;+g \end{array}$$ (4)

is solved by a simple Linear Regression using the Broyden–Fletcher–Goldfarb–Shanno (BFGS) algorithm. The final weights for the PCs can also be referred to as individual shape parameters.

## 3 Evaluation

### 3.1 Accuracy and computational expenses

To measure the runtime and accuracy of our PCAwarp algorithm for a varying number of scalp proxy points and first PCs, we run our PCAwarp algorithm on 50 randomly selected OASIS heads on a machine with an Intel Core i7 CPU (1.7 GHz, 4 cores), 16 GB RAM, running macOS 15.6.1. The algorithms were implemented in Python 3.13 and executed using a single thread. In Table 1, we report mean runtime and median accuracy per surface mesh. The number of scalp proxy points are 21, 64, and 343 and are derived from standard EEG 10-20, EEG 10-10, and EEG 10-5 electrode layouts, respectively.

**Table 1.** Runtime and median shape differences by number of PCs and electrodes.

			Average shape difference [mm]			
Number of PCs	Number of electrodes	Runtime [sec]	Scalp	Skull	CSF	Cortex
	21	7.4	1.72	1.92	1.92	2.11
1	64	8.8	1.69	1.90	1.88	2.10
	343	12.3	1.69	1.91	1.89	2.11
	21	31.8	1.40	1.64	1.65	1.92
5	64	34.2	1.35	1.58	1.64	1.91
	343	58.7	1.24	1.51	1.60	1.91
	21	72.8	1.12	1.47	1.65	1.98
10	64	77.0	0.98	1.34	1.60	1.92
	343	120.6	0.89	1.32	1.49	1.83
	21	126.3	1.14	1.71	1.74	2.42
16	64	140.2	0.90	1.63	1.77	2.34
	343	241.2	0.80	1.40	1.57	2.19
	21	559.2	1.03	2.27	2.17	2.58
50	64	602.0	0.70	2.26	2.21	2.53
	343	954.4	0.58	2.04	2.07	2.45
	21	2744.4	1.87	2.74	2.40	2.69
316	64	3278.9	0.77	2.63	2.39	2.65
	343	5083.0	0.43	2.56	2.35	2.62

While runtime increases with both a growing number of used first PCs and scalp proxy points, approximation accuracy improves steadily as scalp proxy points increase. However, the number of PCs has a far more substantial influence on the final reconstruction accuracy than the number of scalp proxy points. Once an adequate number of first PCs is chosen, adding more scalp proxy points yields only marginal gains in accuracy. Using a large number of PCs tends to overfit on the scalp proxy points, leading to larger shape difference errors, particularly for skull, CSF and cortex. We, therefore, recommended using 10 to 16 PCs as a good compromise between accuracy and runtime and a reasonable set of scalp proxy points. In practice, more points are beneficial up to a point, but beyond roughly 300–500 points the accuracy gains are minimal while runtime and preparation effort increase. We emphasize that it is essential to distribute the scalp proxy points across the entire head above the ears.

### 3.2 Experiment: Approximate shapes of scalp proxies of 16 subjects

The experiment was designed to test the feasibility and robustness of our algorithm for different head types and qualities of recorded scalp proxy data. Therefore, 13 photogrammetry scans (Setup A) and three electrode montages (Setup B) were recorded. All experiments involving humans were conducted with approval from the Ethics Committee of the TU Berlin, following the Declaration of Helsinki.

#### 3.2.1 Setup A

To test the feasibility of approximating the individual head geometry by photogrammetric scans of the outer head shape, 13 Caucasian subjects (mixed male and female, aged 23 to 59) using a *Shining 3D EinStar 3D-Scanner*. The workflow for the 13 subjects was oriented on the FieldTrip (Oostenveld et al., 2011) tutorial on *Localizing electrodes using a 3D-scanner*^2^ (Homölle & Oostenveld, 2019) and is described in detail in the following:

- Measure the subject’s head circumference from NAS via RPA, inion (IZ), LPA, and back to NAS with a flexible measuring tape. Select a cap from the closest cap size (54 cm, 56 cm, 58 cm, or 60 cm) and attach it to the subjects without electrodes. Ensure that the cap is fitted tightly enough to minimize hair convexities.
- Scan the subject’s head following the instructions of the *Shining 3D* recording software. The scans took approximately 2–5 min each, and the subject was instructed to sit still. Export the scan as.obj file.
- In a mesh manipulation software such as MeshLab^3^, manually crop the mesh so it only contains the cap and head without everything below the ears and resave the cropped mesh as an.obj file.
- Load (*ft_read_headshape*) and plot (*ft_plot_headshape*) the cropped mesh in Matlab and manually detect the fiducials (NAS, LPA, RPA) or read their coordinates out if having marked them on the subject before scanning.

The resulting mesh vertices and fiducials were loaded into our individualization algorithm PCAwarp^4^, cut above the ears (35 mm above the plane spanned by the connecting vectors between NAS, LPA, and RPA), and decimated to around 150 vertices. These vertices were used as a scalp proxy, from which the individual surface meshes were computed using our PCAwarp method. The wMNI-4 individualization algorithm (Acar & Makeig, 2013; Darvas et al., 2006) was also calculated using this scalp proxy.

For comparison, an already existing, maximally 3-year-old T1-weighted MRI scan of each subject was requested. This T1 scan was segmented using the same automatic pipeline, and the four resulting meshes for the scalp, skull, CSF, and cortex were used as ground truth against which the errors were calculated. Additionally, Colin27 and the ICBM-152 were segmented in this manner. In addition to the PCAwarp, wMNI-4, Colin27, and ICBM-152 head models, we compared the results to two further head models, referred to as “PCAwarp (perfect scalp)” and “wMNI-4 (perfect scalp)”. These two are constructed by running the PCAwarp and the wMNI-4 individualization algorithm on the original, cut above the ears, scalp mesh of the MRI segmentation, mimicking a perfectly scanned head surface.

#### 3.2.2 Setup B

As an alternative challenge, for three additional subjects, we use 64 electrode positions picked from a 3D scan performed with a smartphone from 2016. We did not anticipate reaching the highest possible accuracy of some of the existing hardware here. However, we hypothesized that the resulting head model would still be an improvement over head model templates even with simple digitization hardware available. Three Caucasian subjects (male, aged 27 to 55) wearing an EEG 10-10 cap were 3D-scanned using an iPhone SE 1st generation (2016, one 12-megapixel camera) and the photogrammetry app *Display.land*^5^. *Display.land* was developed by Ubiquity6 and targeted the gaming community, as it enables users to 3D-scan objects and utilize them during game design and development. The workflow for three subjects was oriented on the FieldTrip (Oostenveld et al., 2011) tutorial on *Localizing electrodes using a 3D-scanner*^6^ (Homölle & Oostenveld, 2019) and is described in detail in the following:

- Measure the subject’s head circumference from NAS via RPA, inion (IZ), LPA, and back to NAS with a flexible measuring tape. Select an EEG cap with the closest cap size (54 cm, 56 cm, 58 cm, or 60 cm) and attach it to the subject without inserting electrodes into the electrode holders. Ensure that the electrode holders along the posterior-anterior midline are in a straight line from NAS to IZ. The electrode holder of Cz should be positioned exactly halfway along the straight line from the nasion to the inion and from the left to the right preauricular points. If necessary, correct and reevaluate until the EEG cap is applied correctly.
- Start the *Display.land* app on the iPhone SE and scan the subject’s head following the *Display.land* instructions. The scan took approximately 4–5 min, and the subject was instructed to sit still. Export the scan as a.obj file.
- In a mesh manipulation software such as MeshLab^7^, manually crop the mesh so it only contains the EEG cap and head without everything below the ears and resave the cropped mesh as a.obj file.
- Load (*ft_read_headshape*) and plot (*ft_plot_headshape*) the cropped mesh in Matlab and manually detect the 64 electrode positions and the fiducials (NAS, LPA, RPA) or read their coordinates out if having marked them on the subject before scanning.
- Since the electrode holder positions had a thickness of 12 mm, a 12 mm inward shift of the electrode positions was applied according to their normals (*moveinward*).

The resulting electrode positions were used as a scalp proxy to compute the PCAwarp and wMNI-4 heads and also compared to the PCAwarp (perfect scalp), wMNI-4 (perfect scalp), ICBM-152, and Colin27.

#### 3.2.3 Results

**Fig. 4.**
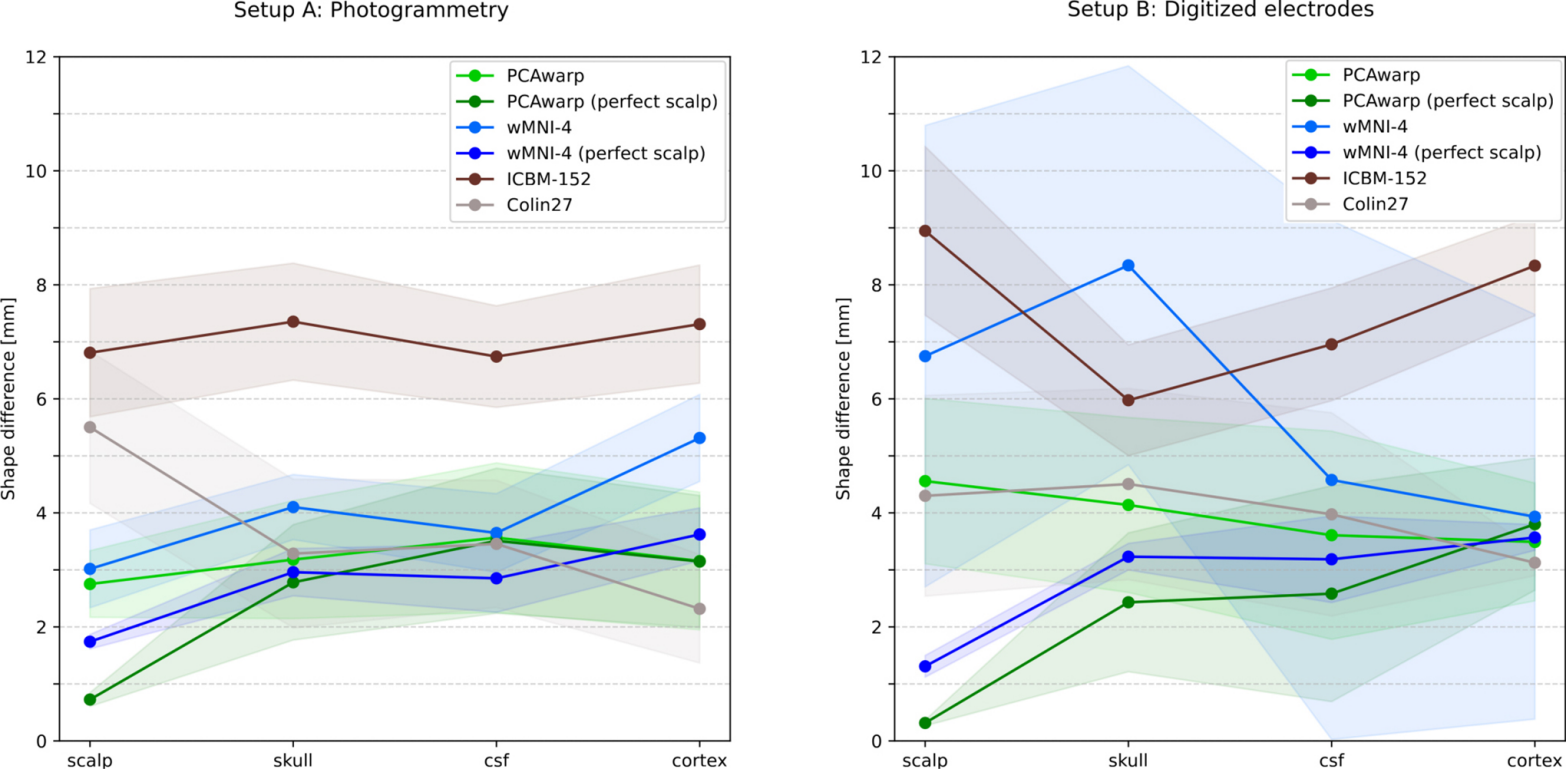
Median shape approximation error and standard deviation shaded of the different head model surface meshes (PCAwarp, PCAwarp (perfect scalp), wMNI-4, wMNI-4 (perfect scalp), ICBM-152, Colin27) from the ground truth scalp meshes measured in vertex distance (left) and shape difference (right). In the upper row, the results are shown for the 13 subjects of Setup A (Photogrammetric scans of scalp surface) and in the lower row for the three subjects of Setup B (digitized electrode positions using a smartphone).

The shape approximation errors for the 13 photogrammetric head scans and the three digitized electrode montages are shown on the left and right of Figure 4, respectively.

For both setups, the PCAwarp surface meshes show lower shape differences to the ground truth surface meshes gained from MRI segmentation than the standard heads ICBM-152 and Colin27 and the wMNI-4 warped surfaces. Assuming to perfectly know the scalp surface, defined here as the ground truth scalp mesh cut above the ears, the PCAwarp (perfectly scalp) and wMNI-4 (perfect scalp) meshes are, as expected, closer to the ground truth than those from the manually scanned scalp.

It is also noteworthy that the individualized head anatomies (PCAwarp, PCAwarp (perfect scalp), wMNI-4, wMNI-4 (perfect scalp)) often exhibit smaller shape differences in the scalp than in the other meshes due to overfitting. This is especially observable for those methods using the scalp proxy from the original MRI (perfect scalp). The methods using the manually digitized scalps (PCAwarp, wMNI-4) show larger shape differences in the scalp due to uncertainties stemming from the manual digitization procedures, in particular, for the smartphone scans (Setup B).

In general, we observed that using the photogrammetric scans (Setup A) yielded better results than using the digitized electrode positions (Setup B) as scalp proxies for both individualization algorithms PCAwarp and wMNI-4.

For the cortex, Colin27 achieved the lowest median difference to the original, closely followed by the other heads except the ICBM-152. Generally, the ICBM-152 shows the most considerable shape differences with 5mm to 9mm for all tissues, due to its large circumference.

**Fig. 5.**
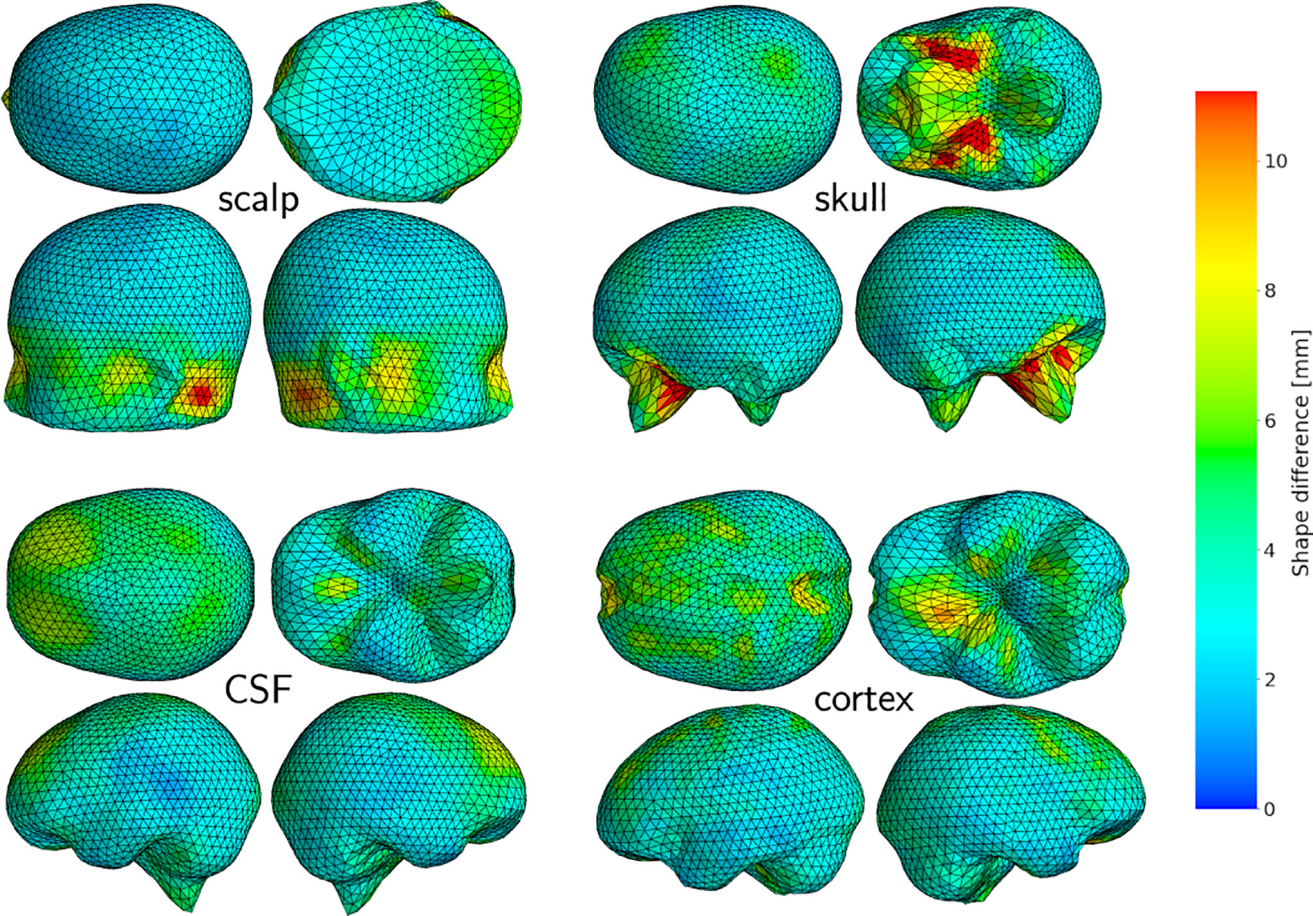
Locally resolved shape difference between ground truth and PCAwarp in mm averaged over the individualized surface meshes of the three subjects shown for the four surfaces: scalp (upper left), skull (upper right), CSF (lower left), and cortex (lower right). Larger errors (∼10 mm) occur close to the nose, ears and the skull’s jaw. The largest errors (∼17.5 mm) are located in the skull mesh close to the brainstem. For improved visibility, values larger than 11 mm were capped to 11 mm.

The average shape difference of the PCAwarped individualized meshes was also plotted on each shell (Fig. 5) to see if all areas are fitted equally well. Generally, the meshes are closest to the ground truth in the anterior regions (<2.5 mm) and slightly less close (>5 mm) in the posterior regions. Larger errors (∼10 mm) are found in regions of rather complex anatomy, such as the ears and nose of the scalp and the skull’s jaw. The most substantial deviation (∼17.5 mm) appears only very locally at the bottom of the skull mesh (brainstem).

We demonstrated that our approach provides spatially better approximated head model anatomies when using photogrammetry scans of the head surface. Even simple electrode digitization using a smartphone delivered head anatomies closer to the original head. In the following, we demonstrate that this improvement in anatomy also yields better source localization results in the EEG inverse problem.

### 3.3 Simulation study: Source localization comparison

Since the final application of our novel head model approximation algorithm is motivated by the aim of providing better forward models for source localization, a simulation study was conducted to measure the improvement in source localization accuracy in source space.

#### 3.3.1 Simulating EEG scalp patterns

For the simulation setup, 22 randomly selected individuals from the OASIS1 database were chosen. The segmentation was again performed using the automatic segmentation pipeline as described above, with the addition of tetrahedralization to derive the FEM volume meshes, which invokes TetGen (Si, 2015) via the iso2mesh wrapper (Fang & Boas, 2009). In each head, 100 random realistic EEG source positions (neocortex, oriented orthogonal to the surface (Buzsáki et al., 2012)) were extracted (for details, please refer to the MRIsegmentation pipeline above).

##### 3.3.1.1 FEM simulation

At each point, an equivalent current dipole was placed to simulate bunches of parallel active neurons since source localization algorithms are often applied to scalp patterns of independent components (ICs) gained from Independent Component Analysis (ICA). These scalp patterns often look *dipolar* such that a single equivalent current dipole is a good approximation (Delorme et al., 2012). The dipolar moments are oriented according to their outwards pointing mesh normals (Dale & Sereno, 1993; Malmivuo & Plonsey, 1995). The EEG potentials at 64 sensors (EEG 10-10 standard locations) were computed using DUNEuro (Schrader et al., 2021) as an FEM solver with the Venant sourcemodel. The final meshes have around 1 million vertices and six tissue types (scalp, skull, CSF, GM, WM, air). The conductivities were fixed at the following values: scalp 0.465 S ${\text{m}}^{-1}$ , skull 0.01 S ${\text{m}}^{-1}$ , CSF 1.65 S ${\text{m}}^{-1}$ , GM 0.276 S ${\text{m}}^{-1}$ , WM 0.126 S ${\text{m}}^{-1}$ , air $2.5\cdot 10^{-14}$ S ${\text{m}}^{-1}$ (Huang et al., 2013). Measurement noise, in the form of white noise, was added to the simulated EEG potentials. Testing three scenarios, we investigated no noise, a signal-to-noise ratio (SNR) of 10 and of 1.

##### 3.3.1.2 Source localization

The source localization was performed using 4-shell BEM head models of the different anatomies. Besides the ground-truth head model (original), we used the ICBM-152, Colin27, and the approximated head models (PCAwarp and wMNI-4). The PCAwarp reconstruction was performed by assuming that the scalp mesh of the subject above the ears had been scanned. The upper scalp vertices of the ground-truth scalp mesh are used as scalp proxies for the fitting algorithms. The PCA was applied only on the remaining 315 heads (leave-one-out cross-validation), and its 315 PCs were used for the PCAwarp reconstruction. For the wMNI-4 reconstruction, the same scalp proxies were used.

All head models are constructed from four surface meshes: scalp, skull, CSF, and cortex, each consisting of 1922 vertices and 3990 triangles. The conductivities for all head models were fixed to scalp 0.465 S ${\text{m}}^{-1}$ , skull 0.01 S ${\text{m}}^{-1}$ , CSF 1.65 S ${\text{m}}^{-1}$ , and cortex 0.201 S ${\text{m}}^{-1}$ (mean of gray and white matter). All head models are in the CTF coordinate system. The simulated potentials at the 64 electrodes of the 4-shell-BEM of the original head anatomy with those of the *ground truth* FEM head model plus noise have a correlation of 0.51±0.29 .

The different head models and linear and nonlinear fitting routines, as described below, are subsequently applied to the simulated FEM potentials with noise. During the iterative fitting process, the sources’ location and orientation were adjusted to maximize the variance in the data explained by the head model. The error used was thus the relative residual variance (RV), which is the relative amount of variance in the data unexplained by the model. The RV is a sum-of-squares type error function that computes the relative deviations of an estimated pattern $\hat{x}$ from the target pattern x:

$$RV=\frac{\sum \;{\left(x-\hat{x}\right)}^{2}}{\sum \;x^{2}}$$ (5)

The RV has the advantage of being normalized to the average amplitude of the measured signal. Two inverse fitting routines were used successively to fit the most optimal location and orientation to a measured scalp pattern: linear search and nonlinear search based on FieldTrips *ft_dipolefitting* (Oostenveld et al., 2011).

- **linear search** compares the RV of the potentials at all electrodes (x) with the computed head model leadfields ($\hat{x}$ ) using OpenMEEG (Gramfort et al., 2010) for every source point in a gridded sourcemodel. This gridded sourcemodel is constructed by placing sources at a regular grid within the cortex mesh of 10 mm distance between adjacent source points in each dimension. The source position, combined with an orientation that yields the lowest RV with the scalp pattern, ultimately constitutes the linear result of the inverse problem.
- **nonlinear search** achieves a further optimized fit by allowing an error minimization algorithm to vary orientation and source position using the linear search result as a starting point.

#### 3.3.2 Results

**Fig. 6.**
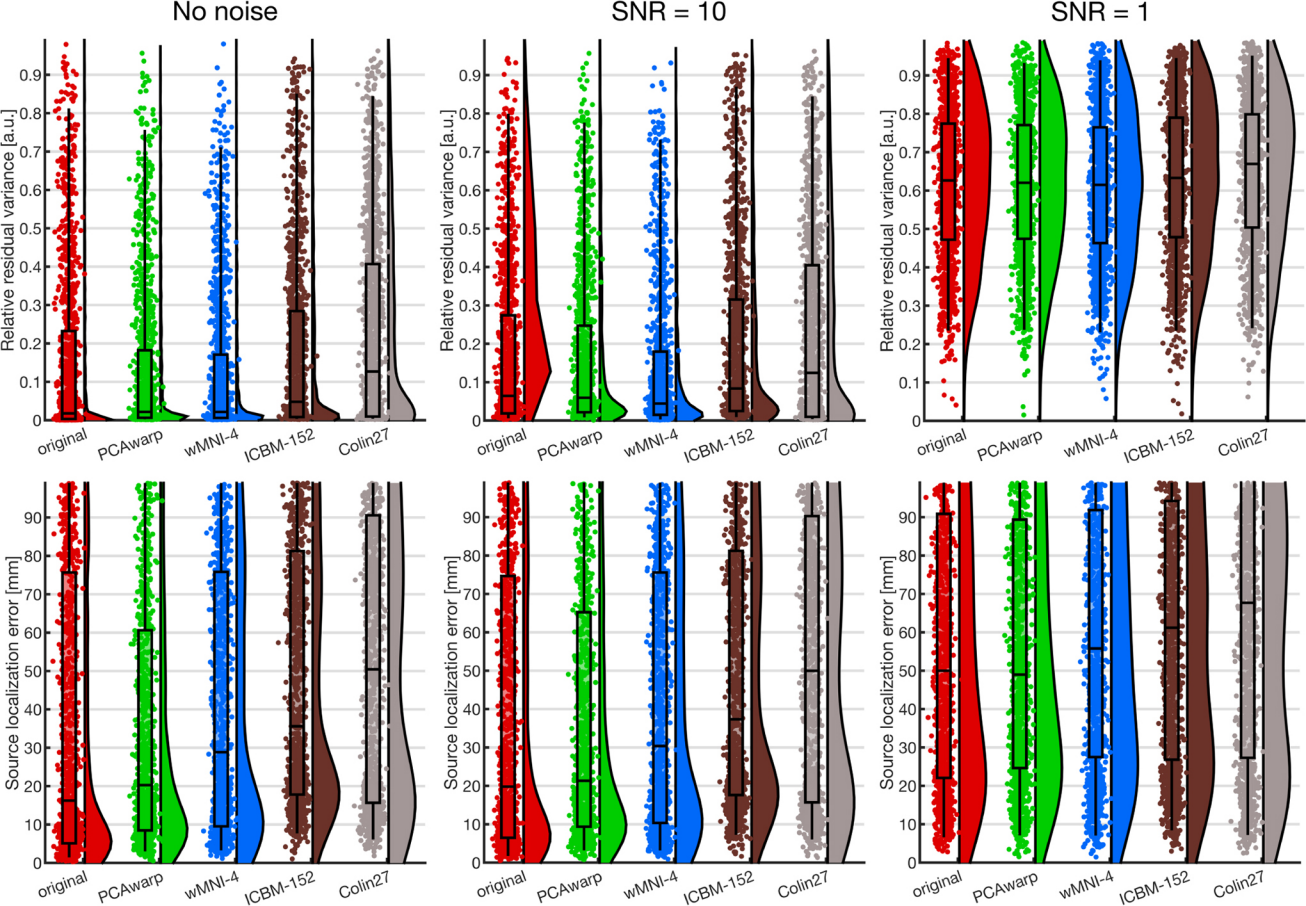
Source localization error of the *ground truth FEM scalp potentials* of the 2200 equivalent current dipoles (22 heads à 100 dipoles) measured in RV in channel space (top row) and Euclidean distance in source space (bottom row) without noise (left column), with noise of SNR=10 (middle column) and SNR=1 (right column). The error is computed with the nonlinear fitting routine for the different head models (original, PCAwarp, wMNI-4, ICBM-152, Colin27) in the original individual head space. Dipoles of with an unsuccessful fit (RV==1.0) were left out for all models and noise levels. In channels space (top row), all head models show low median RVs, where the wMNI-4 performed slightly best (not significant). In source space (bottom row), the PCAwarp method is slightly better than the wMNI-4 method and very close to the original head model. The ICBM-152 and Colin27 perform worse in both channel and source space.

In case of an unsuccessful fit, that is, RV == 1.0 (aborted dipole fitting routine), the scalp pattern and its dipole were discarded from the analysis for all head models and noise levels. As error measure, we compute the inverse fitting error as measured in RV in channel space (Fig. 6 top) and Euclidean distance in source space (Fig. 6 bottom). This source space is in the individual CTF coordinate system of the 22 heads. Therefore, the estimated source positions of the ICBM-152 and Colin27 are translated into the individual CTF coordinate system using the nonlinear transformations derived by the SPM12 MRI segmentations.

Across all SNR conditions, all pairwise model comparisons of the source localization error are significant with p<0.05 (one-sided Wilcoxon) after Benjamini–Hochberg false discovery rate correction, except the comparisons between original and PCAwarp (p = 0.07 (nonoise), p = 0.25 (SNR=10), p = 0.60 (SNR=1)) and for the SNR=1 szenario the comparisons of wMNI-4 with original (p = 0.06), ICBM-152 (p = 0.09), PCAwarp (p = 0.09).

Across all noise levels, the median source localization errors in source space are relatively large (>15 mm) for all head models, even for the original head anatomy. The smallest median error in source space is achieved with the noise-free data by the original head model (16.2 mm), closely followed by our PCAwarp (20.3 mm) and the wMNI-4 (28.8 mm). The fits of the ICBM-152 (35.6 mm median localization error) and Colin27 (50.4 mm median localization error) are much worse. Noteworthy is that this result is not reflected in RV in channel space. The original head model performs best in median RV (0.018 ), closely followed by the PCAwarp and the wMNI-4 method (both 0.022 ). The median RV of the ICBM-152 and Colin27 are larger (0.048 and 0.127 , respectively).

With little additive noise (SNR=10), the results in source space do not differ much from the noise-free scenario. With substantially more noise (SNR=1), all source localization errors drastically increased and reached 50 mm to 67 mm. This is also reflected in large median RVs of 0.61 to 0.67.

## 4 Discussion

We have developed a new approach to approximating individual head geometries using scalp proxies. We demonstrated that the resulting head models significantly enhance source localization compared to standard head models and other individualization approaches.

The approach was tested twofold against the commonly used ICBM-152 and Colin27 and the warped ICBM-152 anatomy (wMNI-4) individualization approach: First, in acquiring photogrammetric scalp scans and digitized electrode positions of a total of 16 subjects using two different hardware modalities and computing the shape differences to the real MRI-based head anatomies. Second, in a simulation study of 22 heads, the scalp surfaces were assumed to be derived by photogrammetry scanning. Realistic EEG scalp patterns with different noise levels were computed for random dipoles within the neocortex by a more detailed FEM model. The source localization errors of the inverse nonlinear fits to the original dipole positions were then computed for the different head models.

In sum, our method (PCAwarp) is closest to the ground truth head geometry in shape difference (Fig. 4) and source localization error in source space (Fig. 6 bottom) across different noise levels. The original head geometry, as extracted from an MRI scan, achieved only slightly better results. The individualized wMNI-4 method is slightly but significantly worse in terms of shape difference and source localization error, while having good RV values, which is the metric used by the inverse fitting algorithm, but less important for source localization accuracy. The ICBM-152 and Colin27 performed worst.

The source localization errors in our simulation study (Fig. 6) are generally larger than those in other studies, for example, by Acar and Makeig (2013) (considerably smaller than 20 mm), because they often use less realistic simulated EEG data using either the same forward and inverse model or simulating the EEG signals without noise. In contrast, we measured a relatively small absolute correlation of the simulated potentials with the ground-truth BEM head model of 0.51±0.29 . This small correlation is caused by the differences in anatomy accuracy (WM/GM accuracy and smoothed surfaces), the use of different solvers (BEM vs. FEM), non-identical electrode positions, and the added noise to the ground truth FEM potentials. We consciously chose this large difference of simulated and inverse modeling data to mimic reality closely. Quick preliminary tests using the 4-shell BEM of the original head anatomy, without noise as ground truth data, showed overall smaller source localization errors, comparable to those reported by Acar and Makeig (2013). It is also noteworthy that the numerical accuracy of the forward problem generally decreases when dipoles are close to the surfaces (Gramfort et al., 2011). Our attempted realistic equivalent current dipoles are chosen to reside in the neocortex quite close to the CSF and the skull surface, hence adding to the overall large localization errors.

The experimental feasibility tests on a total of 16 subjects, which approximated individual head geometry using photogrammetry scans of the outer head shape (Setup A) and simple electrode digitization (Setup B), were both successful, even with a smartphone camera from 2016. However, the approximation error was smaller when using more professional 3D scanning hardware, such as photogrammetry, which has a better scanning resolution and also provided (in our setup) more scalp proxy points, which also improves shape approximation accuracy (Table 1). The final improvement by more accurate scalp proxy scanning was, however, shown to be limited by the results obtained using the original scalp surface for reconstruction (Fig. 4, “perfect scalp”).

The scalp surface exhibited the smallest shape approximation error due to overfitting on the scalp proxy points. This tendency was shown in Table 1 to increase with the use of an increased number of PCs during the warping procedure. 10 to 16 PCs are good tradeoff between scalp overfitting, runtime and shape accuracy. Largest shape approximation errors were detected to reside close to the nose and ears, the skull’s jaw and the brainstem (Fig. 5).

Generally, we observed that our reconstruction algorithm is more robust than the wMNI-4 warp, which sometimes produced distorted and rather unnatural head shapes that sometimes needed manual selection of scalp proxy points to improve the reconstruction. A similar observation was made for the implementation of the wMNI-4 warp in Brainstorm (Tadel et al., 2011). Our warping method already produces excellent results for very few scalp proxy points (21 electrodes, Table 1). However, problems occur if the scalp proxy points are not well distributed over the upper scalp above the ears. As the number of scalp proxy points increases, the shape approximation accuracy improves at the cost of an increased runtime, reaching 2 to 4 min for 10 to 16PCs.

We also investigated the influence of different hairstyles on the results. The results of a bold subject to those of subjects with thick, curly hair were not significantly better, though the standard deviation was smaller. We also observed that short, off-standing hair was sometimes even harder to depress compared to long hair. People with plait tend to have a larger deviation in the back of the head, which was again not significant. Moreover, these differences were only observable in the scalp mesh; the morphed skull, csf, cortex showed even less influence on the hairstyle.

The authors were also unable to test for any performance differences between different races, yet. Nevertheless, we are currently working on constructing similar PCA databases for other races than Caucasian, as well as children’s and infant heads, to be added to the public repository.

We also see some room for improvement in our PCAwarp algorithm. For instance, by increasing the number of head anatomies used for training or by using more complex machine-learning techniques to extract the low-dimensional space and also to estimate the individual head anatomies from a given scalp proxy. Moreover, we plan to extend the PCAwarp algorithm to the neck part, enabling the use of HArtMuT (Harmening et al., 2022), that is, including ocular and muscular artefacts into the source localization process.

To summarize, using data-driven individualized head models based on knowledge of the subject’s scalp geometry can significantly improve source localization accuracy if MRI scans are unavailable. Therefore, we believe that our head model individualization algorithm has the potential to enhance M/EEG source localization accuracy in many future studies with minimal additional effort, facilitating easy adoption by the community.

## Data Availability

The code for the PCAwarp individualization algorithm is available at https://github.com/harmening/headmodel_individualization. The data that support the findings of this study are available on reasonable request from the corresponding author. They are not publicly available because they contain information that could compromise the privacy of research participants.

## Footnotes

1 A typical problem was a too sharp peak of the mesh at the brainstem’s position. Vertices located too far away from neighboring vertices were smoothed by relocation to the geometric mean of neighboring vertices.

2
https://www.fieldtriptoolbox.org/tutorial/electrode/

3
https://www.meshlab.net/

4
https://github.com/harmening/headmodel_individualization

5 https://display.land/, sunset in August 2020. Tested alternatives are for example *Scaniverse* and *Scandy Pro*.

6
https://www.fieldtriptoolbox.org/tutorial/electrode/

7
https://www.meshlab.net/
